# Correcting Mortality for Loss to Follow-Up: A Nomogram Applied to Antiretroviral Treatment Programmes in Sub-Saharan Africa

**DOI:** 10.1371/journal.pmed.1000390

**Published:** 2011-01-18

**Authors:** Matthias Egger, Ben D. Spycher, John Sidle, Ralf Weigel, Elvin H. Geng, Matthew P. Fox, Patrick MacPhail, Gilles van Cutsem, Eugène Messou, Robin Wood, Denis Nash, Margaret Pascoe, Diana Dickinson, Jean-François Etard, James A. McIntyre, Martin W. G. Brinkhof, for IeDEA East Africa, West Africa and Southern Africa

**Affiliations:** 1 Institute of Social and Preventive Medicine (ISPM), University of Bern, Switzerland; 2 Moi University School of Medicine, Eldoret, Kenya; 3 Lighthouse Trust, Kamuzu Central Hospital, Lilongwe, Malawi; 4 Division of HIV/AIDS, Department of Medicine, University of California, San Francisco, San Francisco, California, United States of America; 5 Center for Global Health and Development, Boston University, Boston, Massachusetts, United States of America; 6 Right to Care, Themba Lethu Clinic, Helen Joseph Hospital, Johannesburg, South Africa; 7 Khayelitsha Médecins sans Frontières programme, University of Cape Town, Cape Town, South Africa; 8 Centre de Prise en Charge, de Recherche et de Formation sur le VIH/SIDA, Abidjan, Côte d'Ivoire; 9 Desmond Tutu HIV Centre, Cape Town, South Africa; 10 Mailman School of Public Health, Columbia University, New York, New York, United States of America; 11 Newlands Clinic, Harare, Zimbabwe; 12 Independent Surgery, Gaborone, Botswana; 13 Institut de Recherche pour le Développement/UMR 145, Montpellier, France; 14 Perinatal HIV Research Unit, Soweto, South Africa; University of Pennsylvania School of Medicine, United States of America

## Abstract

**Background:**

The World Health Organization estimates that in sub-Saharan Africa about 4 million HIV-infected patients had started antiretroviral therapy (ART) by the end of 2008. Loss of patients to follow-up and care is an important problem for treatment programmes in this region. As mortality is high in these patients compared to patients remaining in care, ART programmes with high rates of loss to follow-up may substantially underestimate mortality of all patients starting ART.

**Methods and Findings:**

We developed a nomogram to correct mortality estimates for loss to follow-up, based on the fact that mortality of all patients starting ART in a treatment programme is a weighted average of mortality among patients lost to follow-up and patients remaining in care. The nomogram gives a correction factor based on the percentage of patients lost to follow-up at a given point in time, and the estimated ratio of mortality between patients lost and not lost to follow-up. The mortality observed among patients retained in care is then multiplied by the correction factor to obtain an estimate of programme-level mortality that takes all deaths into account. A web calculator directly calculates the corrected, programme-level mortality with 95% confidence intervals (CIs). We applied the method to 11 ART programmes in sub-Saharan Africa. Patients retained in care had a mortality at 1 year of 1.4% to 12.0%; loss to follow-up ranged from 2.8% to 28.7%; and the correction factor from 1.2 to 8.0. The absolute difference between uncorrected and corrected mortality at 1 year ranged from 1.6% to 9.8%, and was above 5% in four programmes. The largest difference in mortality was in a programme with 28.7% of patients lost to follow-up at 1 year.

**Conclusions:**

The amount of bias in mortality estimates can be large in ART programmes with substantial loss to follow-up. Programmes should routinely report mortality among patients retained in care and the proportion of patients lost. A simple nomogram can then be used to estimate mortality among all patients who started ART, for a range of plausible mortality rates among patients lost to follow-up.

*Please see later in the article for the Editors' Summary*

## Abstract 2

Matthias Egger and colleagues present a nomogram and a web-based calculator to correct estimates of program-level mortality for loss to follow-up, for use in antiretroviral treatment programs.

## Editors' Summary

**Background:**

AIDS has killed more than 25 million people since 1981 and about 33 million people (30 million of them in low- and middle-income countries) are now infected with HIV, which causes AIDS. HIV destroys immune system cells, leaving infected individuals susceptible to other infections. Early in the AIDS epidemic, most HIV-infected people died within 10 years of infection. Then, in 1996, highly active antiretroviral therapy (ART) became available. For people living in affluent, developed countries, HIV/AIDS became a chronic condition, but for people living in low- and middle-income countries, ART was prohibitively expensive and HIV/AIDS remained a fatal illness. In 2003, this situation was declared a global health emergency and governments, international agencies, and funding bodies began to implement plans to increase ART coverage in developing countries. By the end of 2009, 5.25 million of the 14.6 million people in low- and middle-income countries who needed ART (36%) were receiving it.

**Why Was This Study Done?:**

ART program managers in developing countries need to monitor the effectiveness of their programs to ensure that their limited resources are used wisely. In particular, they need accurate records of the death (mortality) rates in their programs. However, in resource-limited countries, many patients drop out of ART programs. In sub-Saharan Africa, for example, only about 60% of patients are retained in ART programs 2 years after starting therapy. In many programs, it is not known how many of the patients lost to follow-up subsequently die, but it is known that mortality is higher among these patients than among those who remain in care. Thus, in programs with high dropout rates and poor ascertainment of death in patients lost to follow-up, estimates of the mortality of all patients starting ART are underestimates. In this study, the researchers develop a simple nomogram (a graphical method for finding the value of a third variable from the values of two other variables) to correct estimates of program-level mortality for loss to follow-up.

**What Did the Researchers Do and Find?:**

The researchers' nomogram uses the percentage of patients lost to follow and the estimated ratio of mortality between patients lost and not lost to follow-up to provide a correction factor that converts mortality among patients remaining in care to mortality among all the patients in a program. The researchers first applied their nomogram to the Academic Model Providing Access to Healthcare (AMPATH), a large ART program in Kenya. They used data collected by outreach teams to estimate mortality among the 40.5% of patients lost to follow-up at two AMPATH sites between 1 January 2005 and 31 January 2007. The uncorrected estimate of mortality over this period was 2.8%, whereas the corrected estimate obtained using the nomogram was 9.4%. The researchers then applied their nomogram to 11 other African ART programs. This time, the researchers used a statistical model to provide estimates of mortality among patients lost to follow-up. Mortality among patients retained in care was 1.4% to 12.0% at 1 year; loss to follow-up ranged from 2.8% to 28.7%. The nomogram provided a correction value for mortality among all patients in the ART program of 1.2 to 8.0, which resulted in absolute differences between uncorrected and corrected mortality of 1.6% to 9.8%. The largest absolute difference was in the program with the largest percentage of patients lost to follow-up.

**What Do These Findings Mean?:**

These findings indicate that, in ART programs where a large percentage of patients are lost to follow-up, program-level mortality estimates based on the mortality among patients retained in the program can be substantial underestimates. This bias needs to be taken into account when comparing the effectiveness of different programs, so the researchers recommend that all programs routinely report mortality among patients retained in care and the proportion of patients lost to follow-up. The nomogram developed by the researchers can then be used to estimate mortality among all patients who started ART using a range of plausible mortality rates among patients lost to follow-up. To help program managers make use of the nomogram, the researchers provide a user-friendly web calculator based on the nomogram on the International epidemiologic Databases to Evaluate AIDS (IeDEA) Southern Africa website.

**Additional Information:**

Please access these websites via the online version of this summary at http://dx.doi.org/10.1371/journal.pmed.1000390.

- This study is further discussed in a *PLoS Medicine* Perspective by Gregory Bisson
- Information is available from the US National Institute of Allergy and Infectious Diseases on HIV infection and AIDS
- HIV InSite has comprehensive information on all aspects of HIV/AIDS
- Information is available from Avert, an international AIDS charity on many aspects of HIV/AIDS, including information on the HIV and AIDS in Africa, and on universal access to AIDS treatment (in English and Spanish)
- The World Health Organization provides information about universal access to AIDS treatment, including the 2010 progress report (in English, French and Spanish)
- The International epidemiologic Databases to Evaluate Aids (IeDEA) Southern Africa website provides access to a calculator for correcting overall program-specific mortality for loss to follow-up

## Introduction

The World Health Organization (WHO) estimates that about 4 million people were receiving antiretroviral therapy (ART) in low- and middle-income countries by the end of 2008, with coverage reaching 42% of the estimated 9.5 million in need of ART [1]. Sub-Saharan Africa represented 70% of the estimated treatment need and 73% of the total number of people receiving treatment in low- and middle-income countries at the end of 2008 [1].

The provision of ART in resource-limited settings follows a public health approach, which is characterized by a limited number of regimens and the standardization of clinical and laboratory monitoring [2]. This approach has been shown to result in similar or superior adherence to therapy and similar virological response when compared to industrialized countries [3]–[5]. Loss of patients to follow-up and care is, however, an important problem in resource-limited settings: A systematic review of published retention rates in ART clinics in sub-Saharan Africa showed that the proportion of patients retained 2 years after starting therapy was approximately 60% [6]. Similarly, in a collaborative analysis of patients starting ART in 15 treatment programmes in Africa, Asia, and South America we found that 21% of patients were lost to follow-up 6 months after starting ART [7]. A systematic review and meta-analysis of studies tracing patients lost to follow-up found that these patients experience high mortality [8] compared to patients remaining in care [9].

The successful treatment of individual patients and the monitoring and evaluation of ART programmes both depend on regular and complete patient follow-up. Programmes with high rates of loss to follow-up and poor ascertainment of deaths in patients lost will underestimate mortality of all patients starting ART. For example, standard Kaplan-Meier survival analyses in which follow-up time in patients lost to follow-up is censored at the last visit will be biased because mortality of these patients is assumed to be identical to comparable patients remaining in care. Analyses restricted to patients remaining in care will also underestimate mortality among all patients who started ART. Biased estimates of programme-level mortality hamper the evaluation of single programmes and the comparison between different programmes and settings.

Nomograms are widely used in medicine [10]. They are graphs that allow the approximate graphical computation of a function; placing a line across its several scales immediately solves the formula [11]. We propose a nomogram and a web-based calculator to correct estimates of programme-level mortality for loss to follow-up. We illustrate its use in a case study from Kenya. We show how mortality among patients lost to follow-up can be predicted on the basis of studies that traced patients to ascertain their vital status. Finally, we apply these methods to 11 ART programmes in sub-Saharan Africa and compare uncorrected and corrected estimates of mortality at 1 year to assess the typical bias that is introduced when loss to follow-up is ignored.

## Methods

### Deriving the Nomogram

Mortality of all patients starting ART in a treatment programme over a defined time period is a weighted average of mortality among patients remaining in care and patients lost to follow-up. It depends on the percentage of patients lost, mortality among patients lost to follow-up, and mortality among patients not lost to follow-up. The mortality observed among patients remaining in care can be multiplied by a correction factor *C* to obtain an estimate of programme-level mortality that takes deaths among patients lost to follow-up into account. This correction factor can be obtained from a nomogram. The algebraic derivation of this nomogram is as follows:

Let

*M_U_* =  Uncorrected estimates of programme-level mortality (with censoring of patients lost)
*M_C_* =  Corrected estimate of programme-level mortality, taking deaths among patients lost into account.
*M_NL_* =  Mortality observed in patients retained in care (not lost to follow-up)
*M_L_* =  Mortality estimated in patients lost to follow-up
*r* =  Proportion lost to follow-up

Note that

*M_NL_*, *M_L_*, and *r* need to refer to the same time period (for example the first year of ART).

Then
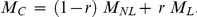
Dividing both sides of this equation by *M_NL_*, we obtain the correction factor
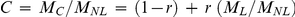
which can be used to obtain *M_C_* for a given *M_NL_* from *M_C_* =  *M_NL_C*.

**Figure 1:**
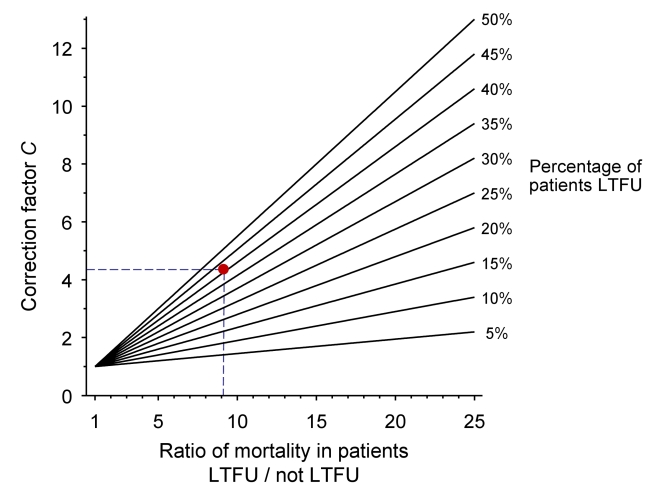
Nomogram for obtaining correction factors to adjust programme-level mortality estimates, based on the observed mortality among patients not lost to follow-up, the observed proportion of patients lost and an estimate of mortality among patients lost. The red dot and broken lines relate to the case study described in Box 1.

The nomogram (Figure 1) plots the ratio of the mortality among patients lost and not lost to follow-up (*M_L_*/*M_NL_*) on lines defined by the proportion of patients lost to follow-up (*r*) which are used to read off correction factor *C*. The broken lines refer to the case-study described in Box 1. Further details on calculations are provided in Text S1. Figures S1 and S2 provide clean versions of the nomogram. These are also available from http://www.iedea-sa.org.

**Box 1. Example from Western Kenya Using Tracing Method**

The Academic Model Providing Access to Healthcare (AMPATH) is a large ART programme in western Kenya [21],[22]. AMPATH is based on a partnership between the Moi University School of Medicine in Eldoret and Indiana University School of Medicine in Indianapolis, Indiana, United States of America. AMPATH provides HIV care and treatment to over 70,000 adults and children living with HIV/AIDS in 18 clinics throughout western Kenya. Patients are managed according to National Kenyan protocols, which are consistent with WHO guidelines. AMPATH undertakes active outreach to patients who miss scheduled appointments (called lost to follow-up in this article). A locator card is completed for all patients enrolling in the programme, which includes contact information and a map to the patient's residence. The results of the outreach programme have been described for the Moi Teaching and Referral Hospital in the city of Eldoret and the rural health centre in Mosoriot [23]. A total of 8,977 adult patients were enrolled in the two participating sites; 3,624 patients were lost to follow-up and 5,353 remained in care between 1 January 2005 and 31 January 2007. Outreach efforts were initiated for 1,143 (31.5%) of patients lost and the vital status of 621 (54.3%) patients could be determined. The naïvely calculated Kaplan-Meier (KM) estimate of programme-level mortality (*M_U_*), ignoring loss to follow-up, was 1.7%. This estimate can now be corrected in six simple steps:

1. Determine the percentage of patients lost to follow-up (*r*):
*3,624 of 8,977 patients were lost to follow-up:* ***40.5%***
2. Determine mortality among patients lost to follow-up (*M_L_*):
*124 of the 621 patients traced had died. The KM estimate was* ***20.0%***
3. Determine mortality among patients not lost to follow-up (*M_NL_*):
*126 of the 5,353 patients remaining in care had died. The KM estimate was* ***2.2%*** *.*
4. Calculate the ratio of mortality among patients lost to follow-up and patients not lost to follow-up (*M_L_* divided by *M_NL_*):
*20.0 divided by 2.2 is* ***9.1***
5. Obtain the correction factor *C* from the nomogram:
***4.3*** *(see broken lines in* *Figure 1* *)*
6. Calculate the corrected programme-level mortality:
*4.3 times 2.2% is* ***9.5%***

Not all patients lost to follow-up could be located and mortality among those who could not be found might have been even higher than in those who were successfully traced. Overall mortality among patients lost to follow-up may thus have been higher than 20%. The effect of assuming a higher mortality among patients lost to follow-up can be examined in a sensitivity analysis: repeating the steps assuming that 25% or 30% of patients lost to follow-up had died results in estimates of corrected programme-level mortality of 11.4% and 13.5%. Clearly, deaths in patients lost to follow-up are an important issue when estimating programme-level mortality in the AMPATH programme.

### Two Ways of Estimating Mortality among Patients Lost to Follow-Up

The parameters *r* and *M_NL_* can be observed directly, but mortality among patients lost to follow-up must be estimated. This can be done in dedicated studies tracing patients lost to follow-up, for example by visiting the homes of patients lost to follow-up or by linking treatment programme with death registry data to ascertain the vital status of patients lost to follow-up [12]–[15]. This method will be henceforth be called “tracing method.”

**Figure 2:**
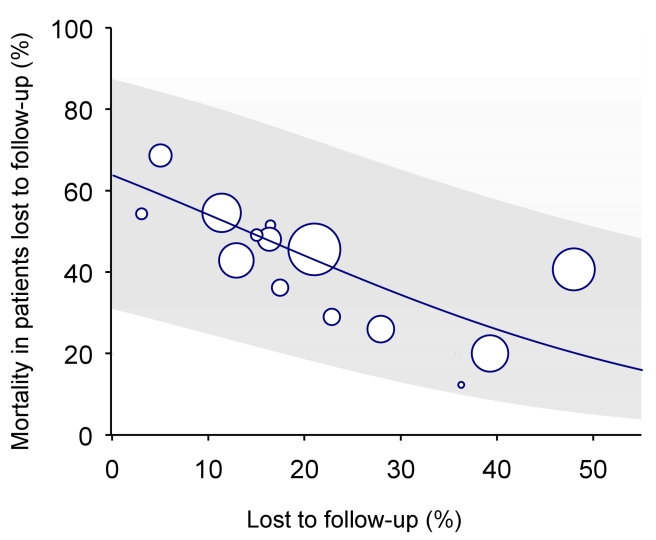
Predicted mortality among patients lost to follow-up according to percent of patients lost in programme (solid line) with 95% CI (limits of grey area). See text for regression equation.

In many situations, however, data from dedicated tracing studies are not available. In this case, mortality among patients lost to follow-up may be predicted on the basis of published data from similar settings. We used Brinkhof and colleagues' systematic review and meta-regression analysis of studies tracing patients lost to follow-up [8]. The meta-regression analysis was based on a total of 15 studies in patients on ART from sub-Saharan Africa. There was an inverse relation between mortality among those lost to follow-up and the rate of loss to follow-up in the programme: The higher the rate of loss to follow-up the lower the mortality among those lost. Figure 2 shows the predicted mortality among patients lost to follow-up according to the percentage of patients lost in the programme, with 95% confidence intervals (CIs). The equation is as follows:
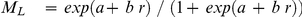
where *a* = 0.57287 and *b* = −4.04409.

Mortality among patients lost to follow-up can be calculated using the formula above or read off Figure 2, based on the proportion of patients lost to follow-up in the programme. This method will henceforth be called “meta method.”

### Allowing for Uncertainty

We used Monte Carlo simulations with 100,000 iterations to calculate 95% CIs for the corrected programme-level mortality (*M_C_*). These simulations allow for uncertainty in the estimation of (i) mortality among patients remaining in care (*M_NL_*); (ii) in the proportion of patients lost to follow-up (*r*); and (iii) of mortality among patients lost to follow-up (*M_L_*). Further details are provided in Text S1.

### Application to ART Programmes in Sub-Saharan Africa

The International epidemiological Databases to Evaluate AIDS (IeDEA, see http://www.iedea-hiv.org) is a collaborative network of HIV/AIDS treatment programmes in seven regions of the world, including North America, Asia and the Pacific, the Caribbean and Latin America, and four regions in sub-Saharan Africa (Central Africa, East Africa, West Africa, and Southern Africa). IeDEA and its predecessor, the ART in Lower Income Countries (ART-LINC) collaboration, have been described in detail elsewhere [16]–[20]. These collaborative networks were established to address clinical and operational research questions that require large patient numbers or many treatment programmes, for example to compare outcomes of ART between different settings, delivery modes, and types of monitoring.

We included 11 ART programmes from sub-Saharan Africa. No direct evidence on the mortality of patients lost to follow-up from tracing studies was available for these programmes, and mortality was therefore predicted using the meta method. All patients aged 16 years or older with complete data on sex and date of birth, and who were treatment naïve at start of combination ART were included in the analysis. Combination ART was defined as a minimum of three antiretroviral drugs from two drug classes. Advanced stage of disease was defined as WHO stages III or IV or Centers for Disease Control and Prevention (CDC) clinical stage C. Measurements of laboratory values closest to the starting date of ART (within 6 months before up to one week after the date of starting ART) were taken as the baseline levels. The data collected at participating sites were cleaned, merged, and analysed centrally. At all sites, institutional review boards had approved the collection and transfer of data.

For each programme we determined the proportion of patients lost to follow-up at 1 year (*r*). A patient was considered lost to follow-up if the last visit was more than 9 months before the closure date for that site, with the closure date defined as the most recent visit date recorded in the database. This allowed for time during which a patient could have returned for a visit. Only patients who potentially had 9 months of follow-up were included in the calculations of *r*. We used Kaplan-Meier methods to estimate mortality (with 95% CI) at 1 year for patients remaining in care (*M_NL_*).

### Web Calculator

The calculations of the nomogram, the prediction of mortality among patients lost to follow-up based on the meta-regression analysis described above, and the calculation of 95% CIs have been implemented on a dedicated website at http://www.iedea-sa.org.

## Results

### Case Study from Western Kenya Using the Tracing Method

Box 1 and Figure 1 illustrate the correction of naïve estimates of programme-level mortality in six simple steps. The data are from the Academic Model Providing Access to Healthcare (AMPATH), a large ART programme in western Kenya [21],[22]. Patients who miss scheduled appointments are actively traced by outreach teams, and data from the outreach programme were used to estimate mortality among patients lost to follow-up. The data are based on a previous detailed analysis of two AMPATH sites [23]. The uncorrected estimate (*M_U_*) of mortality at these two sites for the period between 1 January 2005 and 31 January 2007 was 1.7% (95% CI 1.3%–2.0%), the estimate for mortality among patients not lost to follow-up was 2.2% (95% CI 1.8%–16.6%), and the corrected estimate using the nomogram was 9.5% (Box 1). Entering the data into the web calculator at http://www.iedea-sa.org/ gives a more precise corrected estimate of programme mortality of 9.4%, with 95% CI 8.1%–10.9%. Of note, the results from the nomogram are similar to the corrected estimates that were obtained for these AMPATH sites by Yiannoutsos et al. using more complex statistical methods [23],[24].

### Correction of Mortality in ART Programmes in Sub-Saharan Africa Using the Meta Method

A total of 24,257 patients from 11 ART programmes in 10 countries (Botswana, Côte d'Ivoire, Kenya, Malawi, Rwanda, Senegal, South Africa, Uganda, Zambia, Zimbabwe) were included: 1,363 deaths were observed during the first year of ART. Table 1 shows the characteristics of the 11 programmes. Four programmes were located in South Africa and one programme had sites in six different countries. The number of patients treated at each site ranged from 369 to 4,705 patients. Eight sites were public (government) clinics offering ART free of charge, two were run by a nongovernmental organisation offering free ART, one was a research site offering free ART, and one site was a private clinic operating on a fee-for-service basis. Median age across all programmes was 35 years (interquartile range 30–41 years) and 16,018 patients (66.0%) were women. The median CD4 cell count at baseline ranged from 83 to 156 cells/µL across programmes.

**Table 1:** Characteristics of ART programmes included in the study.

Site	Location	Characteristics	Patients, *n*	Enrolment Period, Calendar Years	Women, *n* (%)	Median (IQR) Age, Years	Median (IQR) Baseline CD4 Cell Count, Cells/µL	Advanced clinical stage, % (95% CI)^a^
ANRS 1215	Dakar, Senegal	Research site, free treatment	369	1998–2002	201 (54%)	38 (31–44)	121 (48–217)	55% (50–60)
CEPREF	Abidjan, Côte d'Ivoire	Public, free treatment	2,643	1998–2007	1,941 (73%)	35 (30–42)	132 (52–217)	81% (80–83)
Independent Surgery	Gaborone, Botswana	Private clinic, fee-for-service	662	1996–2007	393 (59%)	36 (32–41)	118 (53–187)	Not assessed
ISS clinic	Mbarara, Uganda	Public, free treatment	3,713	1996–2007	2,173 (59%)	36 (31–42)	99 (35–181)	81% (79–82)
Lighthouse	Lilongwe, Malawi	Public, free treatment since June 2004	4,705	2004–2007	2,811 (60%)	36 (30–42)	126 (54–211)	86% (85–87)
Newlands	Harare, Zimbabwe	NGO, free treatment	857	1996–2007	585 (68%)	37 (32–44)	102 (51–159)	68% (63–72)
Gugulethu	Cape Town, South Africa	Public, free treatment	1,896	2002–2006	1,294 (68%)	33 (29–39)	103 (50–160)	80% (78–82)
Khayelitsha	Cape Town, South Africa	Public, free treatment	3,366	2001–2005	2,353 (70%)	32 (28–38)	87 (35–146)	90% (89–91)
PHRU	Soweto, South Africa	Public, free treatment	528	2001–2005	373 (71%)	35 (30–41)	83 (33–139)	45% (40–49)
Themba Lethu	Johannesburg, South Africa	Public, free treatment	3,694	1996–2006	2,491(67%)	35 (30–41)	87 (34–152)	97% (96–97)
MTCT-Plus Initiative	Sites in South Africa, Zambia, Kenya, Rwanda, Uganda, Côte d'Ivoire	NGO, family based care, free treatment	1,824	1996–2006	1,403 (77%)	30 (27–35)	156 (93–198)	40% (38–42)

a Defined as WHO stages III or IV or Centers for Disease Control and Prevention (CDC) clinical stage C.

ANRS, Agence Nationale de Recherches sur le SIDA et les Hépatites Virals; CEPREF, Centre de Prise en Charge de Recherche et de Formation; IQR, interquartile range; ISS Immune Suppression Syndrome clinic; MTCT, Mother To Child Transmission; NGO, nongovernmental organisation, PHRU, Perinatal HIV Research Unit.

**Figure 3:**
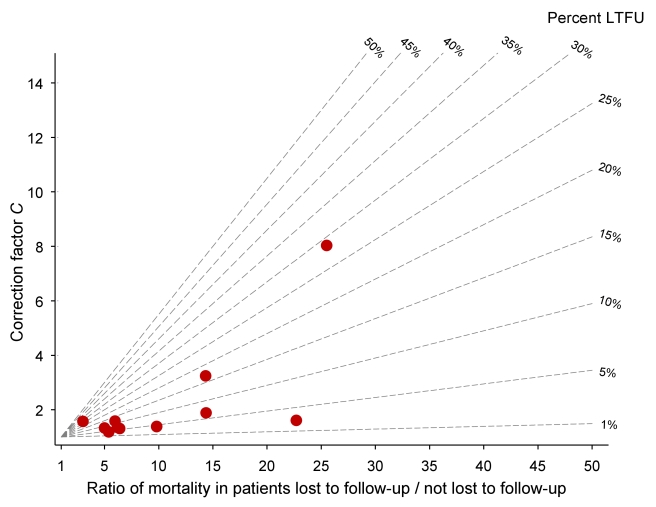
Nomogram with data from 11 antiretroviral treatment programmes in sub-Saharan Africa. LTFU, lost to follow-up.

Table 2 lists the uncorrected estimates (*M_U_*) of programme-level mortality at 1 year (which do not consider mortality among patients lost to follow-up), the proportion of patients lost to follow-up at 1 year (*r*), the estimated 1-year mortality among patients remaining in care (*M_NL_*), the predicted mortality among patients lost to follow-up (*M_L_*, obtained from the meta-regression model of Brinkhof et al. [8]), correction factor *C* and the corrected estimates of programme-level mortality at 1 year (*M_C_*), which take mortality among patients lost to follow-up into account, with 95% CIs calculated as described above. Figure 3 shows the nomogram populated with the data from the 12 antiretroviral treatment programmes.

**Table 2:** Uncorrected Kaplan-Meier estimates of programme-level mortality at 1 year for all patients starting ART, number of patients lost to follow-up, mortality estimates for patients retained in care, predicted mortality among patients lost to follow-up, correction factor *C* and corrected programme-level mortality at 1 year.

Site	Uncorrected Estimates of Mortality (*M_U_*), % (95% CI)	Patients Eligible for Calculation of Loss to Follow-Up^a^, *n*	Patients Lost to Follow-Up, *n* % (*r*)	Mortality among Patients Retained in Care (*M_NL_*), % (95% CI)	Mortality among Patients Lost to Follow-Up (*M_L_*), % (95% CI)	*C*	Corrected Mortality (*M_C_*), % (95% CI)^b^	Difference between Corrected and Uncorrected Mortality, % (*M_C_* − *M_U_*)
A	2.7% (2.0–3.7)	1,132	32 (2.8%)	2.7% (2.0–3.7)	61.3% (29.3–88.8)	1.62	4.4% (3.1–5.7)	1.6%
B	10.8% (8.0–14.5)	369	16 (4.3%)	11.1% (8.2–14.8)	59.8% (28.3–84.8)	1.20	13.3% (10.0–17.1)	2.4%
C	6.0% (4.5–7.8)	656	28 (4.3%)	6.1% (4.6–8.0)	59.9% (28.4–84.9)	1.38	8.4% (6.3–10.7)	2.4%
D	8.9% (8.0–9.9)	2,827	160 (5.7%)	9.1% (8.2–10.2)	58.5% (27.5–84.0)	1.31	11.9% (9.9–13.7)	3.0%
E	9.1% (7.8–10.7)	1,074	70 (6.5%)	9.4% (8.0–11.0)	57.7% (27.0–83.4)	1.33	12.5% (10.1–15.0)	3.4%
F	3.8% (2.6–5.7)	632	42 (6.6%)	4.0% (2.7–5.9)	57.5% (26.9–83.3)	1.89	7.6% (5.1–10.3)	3.7%
G	11.0% (8.5–14.11)	340	28 (8.2%)	11.2% (8.7–14.4)	56.0% (25.9–82.2)	1.33	14.9% (11.4–18.8)	3.9%
H	8.2% (7.2–9.4)	2,212	258 (11.7%)	8.8% (7.7–10.0)	52.5% (23.7–79.7)	1.58	13.9% (10.4–17.3)	5.7%
I	3.0% (2.4–3.6)	3,083	518 (16.8%)	3.3% (2.7–4.0)	47.3% (20.6–75.7)	3.26	10.8% (6.2–15.6)	7.7%
J	10.6% (9.6–11.6)	3,194	904 (28.3%)	12.0% (11.0–13.2)	36.1% (13.8–66.5)	1.59	19.1% (12.5–27.4)	8.2%
K	1.3% (0.09–1.8)	1,942	558 (28.7%)	1.4% (1.0–1.9)	35.7% (13.6–66.2)	8.30	11.6% (4.9–20.0)	9.9%

Eleven antiretroviral treatment programmes in sub-Saharan Africa, ordered by increasing loss to follow-up.

a Patients with at least 9 months of potential follow-up who are at risk of being classified as lost to follow-up.

b The corrected estimates of programme-level mortality with 95% CIs can be obtained from the web calculator available at http://www.iedea-sa.org. The 95% CI are based on Monte Carlo simulations, taking into account uncertainty in mortality among patients lost to follow-up, uncertainty in mortality among patients remaining in care and uncertainty in the proportion of patients lost to follow-up. See Text S1 for further details.

**Figure 4:**
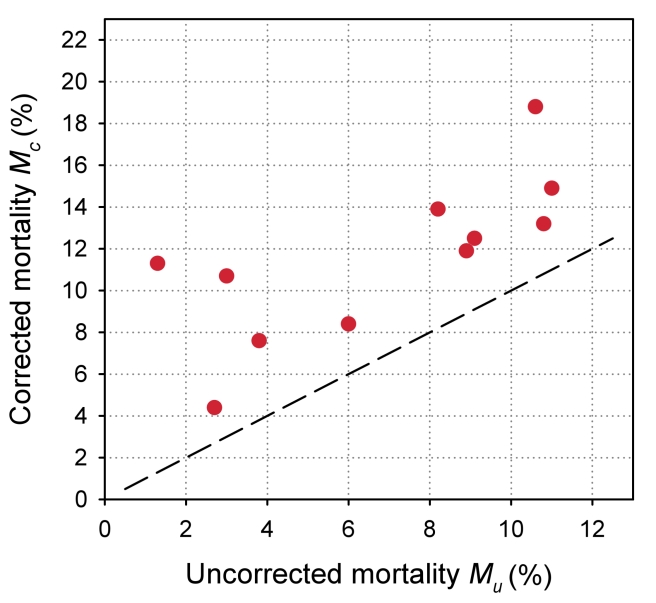
Scatterplot of uncorrected versus corrected mortality for loss to follow-up in 11 treatment programmes in sub-Saharan Africa.

The uncorrected estimates of programme-level mortality at 1 year ranged from 1.3% to 11.0%, mortality at 1 year among patients retained in care from 1.4% to 12.0%; loss to follow-up at 1 year from 2.8% to 28.7%; and correction factor *C* from 1.19 to 8.04. The corrected estimates of programme-level mortality ranged from 4.4% to 18.8%. Figure 4 shows a scatter plot of the uncorrected estimates of programme-level mortality (*M_U_*) against the corrected estimates (*M_C_*): the absolute difference between uncorrected and corrected mortality at 1 year was relatively small (1.7%–3.6%) in seven programmes, but more substantial in the remaining four programmes. The largest difference in mortality was 9.8%, in a programme with 28.7% of patients lost to follow-up at 1 year. The uncorrected estimate of programme-level mortality for this programme was 1.4% and the corrected estimate 11.2%.

## Discussion

Regular and complete patient follow-up is essential both for the care of individual patients and the monitoring and evaluation of outcomes of ART programmes. Individual treatment decisions can then be made in the light of clinical and laboratory results and the rate of complications and mortality can be accurately estimated at the programme level. Loss to follow-up is, however, an important problem in ART programmes in resource-limited settings [6],[7],[18],[25], and poor ascertainment of deaths in patients lost to follow-up may mean that programme-level mortality, i.e. mortality of all patients starting ART, is underestimated [8],[23]. Previous analyses of treatment programmes have generally censored follow-up time at the last visit to the clinic, and patients lost to follow-up therefore contributed follow-up time but no deaths [26]–[28] (here referred to as *M_U_*). We developed a simple nomogram that allows programme managers to read off a correction factor for a range of plausible mortality rates among patients lost to follow-up. This factor can then be used to assess to what extent the mortality observed among patients retained in care underestimates mortality at the programme level.

We applied the nomogram method to ART programmes in sub-Saharan Africa to estimate programme-level mortality at 1 year after starting ART and found that the bias was modest in many programmes, because loss to follow-up was relatively low. It is clear from the shape of the nomogram that if the proportion of patients lost is low (say, below 10%), the correction factor will not be greatly affected by different assumptions for mortality among patients lost to follow-up. In programmes with fewer than 10% of patients lost, the mortality observed among patients retained in care will thus generally provide a reasonable estimate, which will underestimate programme-level mortality by only a few percentage points. Conversely, if a large proportion of patients are lost, as in the case study from western Kenya (Box 1), the bias will be substantial even if the ratio of mortality between patients lost and not lost is relatively low, and the amount of bias will increase steeply with higher ratios.

Mortality among patients lost to follow-up is high: Brinkhof et al. recently reviewed studies that traced patients who became lost to ART programmes in resource-limited settings [8]. The vital status of about two-thirds of patients could be ascertained, and among these many had died: in ART programmes from sub-Saharan Africa the combined mortality from meta-analysis was 46% (95% CI 39%–54%) [8]. Patients were often lost in the first few months of ART and died soon thereafter [8].These findings are in accordance with previous analyses from the ART-LINC collaboration [27] and other treatment programmes, for example the Médecins Sans Frontières programmes in Malawi [28] and South Africa [29], or the ART programme in Mbarara, Uganda [25]. Of note, the percentage of patients lost to follow-up in these programmes was associated with estimated mortality rates in the patients lost: the estimated mortality declined from around 60% to 20% as the percentage of patients lost to the programme increased from 5% to 50%. As discussed in detail elsewhere [8], those lost to follow-up in programmes with high rates of loss to follow-up might include many low-risk patients who self-transferred to another programme, for example because of a more convenient location of the new clinic, to avoid stigma or due to work-related reasons. The negative association between the proportion lost and mortality among patients lost to follow-up will attenuate the effect of a high rate of loss to follow-up on the correction factor *C*.

In the absence of direct evidence, the regression analysis of Brinkhof et al. [8] can be used to predict mortality among patients lost to follow-up for a given programme. The model will provide a sensible range of estimates of mortality, which can then be used in analyses to adjust overall mortality. We used this approach when applying the nomogram to the 11 ART programmes from sub-Saharan Africa. Statistical uncertainty is, however, substantial, as documented by the wide confidence intervals around the predicted mortality among patients lost to follow-up (the grey area in Figure 2), and this translated into wide confidence intervals around the corrected estimates of programme-level mortality (see last column of Table 2).

In addition to the statistical uncertainty, the applicability of the results from the regression analysis must also be considered: The studies examined loss to follow-up and mortality in the first months after starting ART, and at present the regression model should therefore not be used to estimate mortality among patients lost to follow-up later on, for example in the second or third year of ART. The different determinants of loss to follow-up, including “silent transfer” to another programme (without notifying the programme where ART was initiated), financial constraints, and improving or deteriorating health will change with time since starting ART, and mortality among patients lost to programmes will change accordingly. In the present study we focused on loss to follow-up in the first year of ART, and the use of data from the review and meta-regression analysis was therefore appropriate. We stress that patterns of loss to follow-up and associated mortality may also change with calendar years, for example with increasing CD4 cell counts at the start of ART [17].

To obtain accurate estimates of mortality among all patients starting ART, programmes should therefore make an effort to trace patients lost to follow-up and ascertain their vital status. The results from these efforts should be made available to the scientific community so that the regression model can be updated and improved, for the benefit of programmes in settings where tracing of patients lost is not possible. Also note that results of studies tracing patients lost to follow-up can be used to directly correct estimates of mortality in the programme, for example by using double-sampling designs or weighted Kaplan-Meier methods [23],[30]. These methods make strong assumptions, however, and some require expert statisticians.

The nomogram method has the important advantage of being simple and adapted to the field. Among the data used in the nomogram the estimated or assumed mortality among patients lost to follow-up (*M_L_*) will always be associated with the greatest degree of statistical uncertainty and the greatest risk of bias. The estimates of mortality among patients remaining in care (*M_NL_*) might, however, also be biased: some patients will not meet criteria for loss to follow-up and their follow-up will be censored at the last visit. Mortality rates might be higher in these patients than in otherwise identical patients whose follow-up time was not censored. In other words, we cannot exclude the possibility that some degree of “informative censoring” might introduce some bias in our estimate of mortality among patients retained in care. Finally, the nomogram can provide only a sensitivity analysis without formal estimates of uncertainty. We overcame this by creating a web calculator with a user-friendly interface, which calculates 95% CI taking into account the statistical uncertainty in the input parameters of the nomogram.

ART programmes should strive to prevent loss to follow-up: interventions that prevent loss to follow-up in resource-limited settings can improve survival and are cost-effective by international criteria [31]. For example, outreach teams that routinely trace patients, combined with other measures, substantially reduce loss to follow-up [18]. Financial constraints are a common reason for not returning to the clinic [8], and mortality in programmes that charge user fees has been shown to be higher than in those offering free treatment [27]. Decentralization of services, task shifting to lay care providers, longer drug refill periods for stable patients, and provision of transport vouchers for those in need are some of the strategies that could address this issue. Strengthening of referral systems and regular exchange of information between clinics, together with patient education, could increase the recording of transfers and ensure continuity of care. Finally, when assessing outcomes, programmes should routinely report both mortality among patients retained in care at a given point in time and the proportion of patients lost to follow-up at that time. A simple nomogram can then be used to estimate mortality among all patients who started ART.

## Supporting Information

Figure S1 Nomogram for obtaining correction factors to adjust programme-level mortality estimates, based on the observed mortality among patients not lost to follow-up (LTFU), the observed proportion of patients lost and an estimate of mortality among patients lost. Horizontal axis shows ratios from 1 to 15.
(0.13 MB TIF)

Figure S2 Nomogram for obtaining correction factors to adjust programme-level mortality estimates, based on the observed mortality among patients not lost to follow-up (LTFU), the observed proportion of patients lost and an estimate of mortality among patients lost. Horizontal axis shows ratios from 1 to 50.
(0.13 MB TIF)

Text S1 Statistical appendix on the calculation of 95% CIs for corrected programme-level mortality by web calculator at http://www.iedea-sa.org.
(0.04 MB DOC)

## Abbreviations

AMPATH: Academic Model Providing Access to Healthcare
ART: antiretroviral therapy
ART-LINC: ART in Lower Income Countries
CDC: US Centers for Disease Control and Prevention
CI: confidence interval
IeDEA: International epidemiologic Databases to Evaluate AIDS
WHO: World Health Organization
