# Supplementary figures and images for: Correcting Mortality for Loss to Follow-Up: A Nomogram Applied to Antiretroviral Treatment Programmes in Sub-Saharan Africa
Source: PLoS Med. 2011 Jan 18;8(1):e1000390. doi: 10.1371/journal.pmed.1000390 (PMC3022522; doi:10.1371/journal.pmed.1000390)

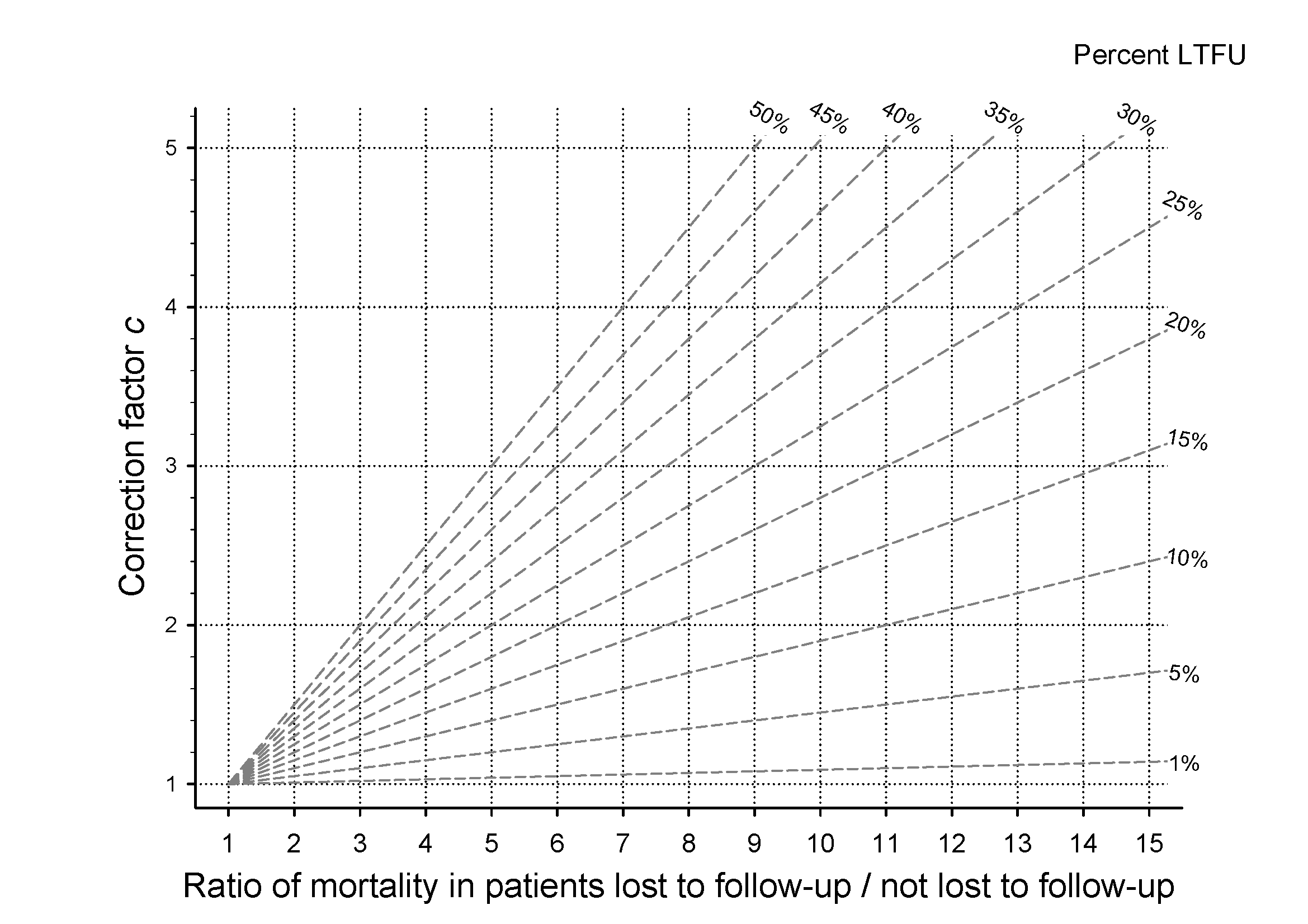

Supplement: Figure S1 — Nomogram for obtaining correction factors to adjust programme-level mortality estimates, based on the observed mortality among patients not lost to follow-up (LTFU), the observed proportion of patients lost and an estimate of mortality among patients lost. Horizontal axis shows ratios from 1 to 15. (0.13 MB TIF) [file pmed.1000390.s001.tif]

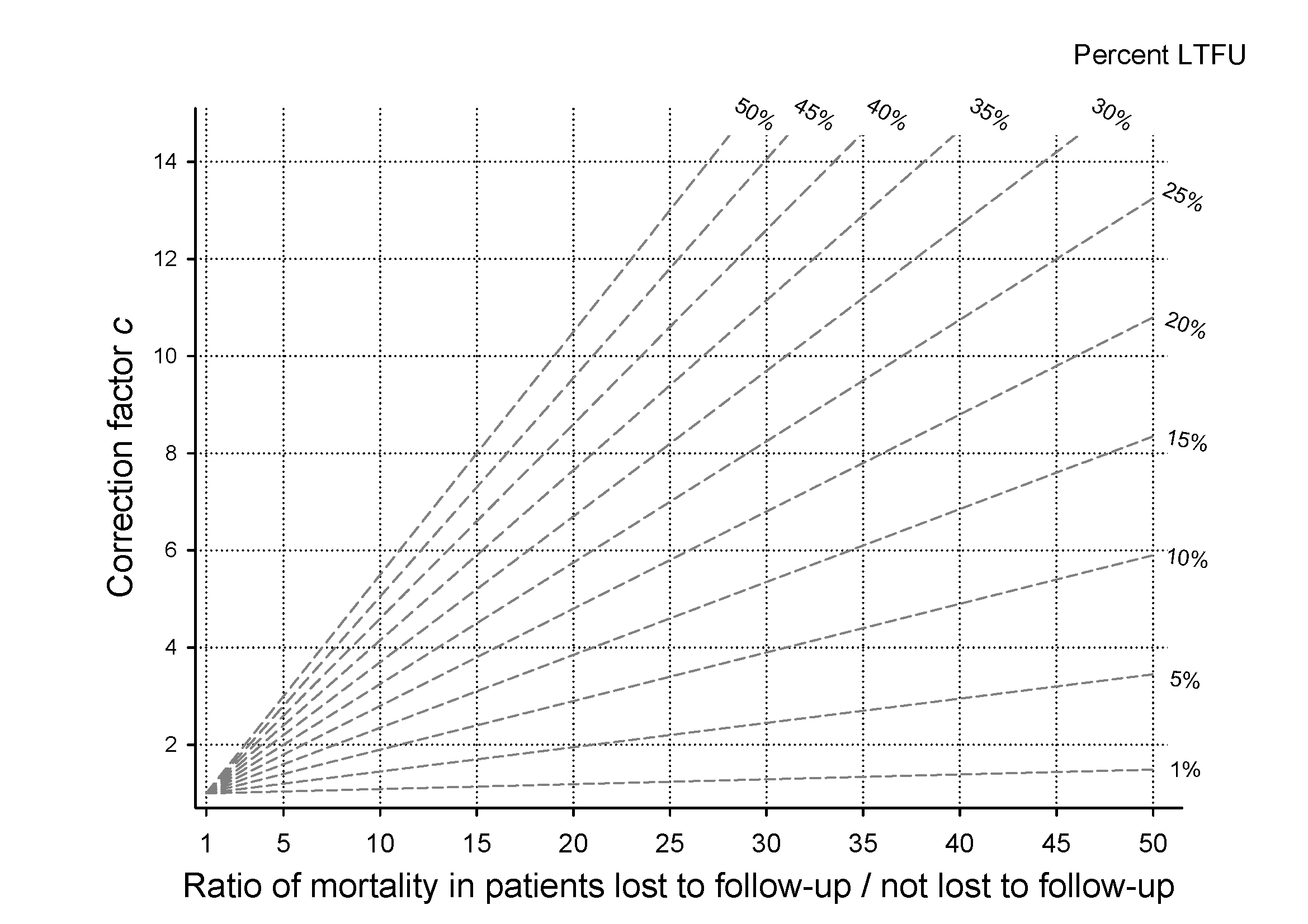

Supplement: Figure S2 — Nomogram for obtaining correction factors to adjust programme-level mortality estimates, based on the observed mortality among patients not lost to follow-up (LTFU), the observed proportion of patients lost and an estimate of mortality among patients lost. Horizontal axis shows ratios from 1 to 50. (0.13 MB TIF) [file pmed.1000390.s002.tif]
