# Supplementary material for: Correcting Mortality for Loss to Follow-Up: A Nomogram Applied to Antiretroviral Treatment Programmes in Sub-Saharan Africa
Source: PLoS Med. 2011 Jan 18;8(1):e1000390. doi: 10.1371/journal.pmed.1000390 (PMC3022522; doi:10.1371/journal.pmed.1000390)
Supplement: Text S1 — Statistical appendix on the calculation of 95% CIs for corrected programme-level mortality by web calculator at http://www.iedea-sa.org. (0.04 MB DOC) [file pmed.1000390.s003.doc]

**Text S1. Statistical appendix on the calculation of 95% confidence intervals for corrected programme-level mortality by web calculator at www.iedea-sa.org.**

We implemented a web calculator that directly calculates the corrected, programme-level mortality with 95% confidence intervals (95% CI) using the two methods presented in the main paper by Egger *et al* (PLoS Medicine 2010): a) the tracing method and b) the meta method. The web calculator can be found at [www.iedea-sa.org](http://www.iedea-sa.org/) (click on tab ‘Tools and calculators’ to get to the web calculator). The user chooses the method to use and then enters the required input values.

In this statistical appendix we provide details on the calculation of the 95% CIs. These are computed using the Monte Carlo method with *k*=100,000 draws, thus allowing for uncertainty in the estimation of i) mortality in patients remaining in care; ii) the proportion of patients lost to follow-up; and iii) mortality in patients lost to follow-up. For i) and iii) sampling is based on the asymptotic normal distribution of log(-log) transformed survival (1-mortality) estimates [1].

Let

*M_NL_* = Mortality observed in patients retained in care (not lost to follow-up)

*S_NL_* = Survival observed in patients retained in care (not lost to follow-up)

*M_L_* = Mortality estimated in patients lost to follow-up

*S_L_* = Survival estimated in patients lost to follow-up

*r* = Proportion lost to follow-up

The steps of the Monte Carlo procedure are as follows:

1. **Tracing method**
   1. Based on estimates of M_NL_ with 95% CI (MNL_UB and MNL_LB for upper and lower bounds, respectively), *k* deviates *M*NL_i (*i* = 1,...,*k*) of mortality among patients remaining in care are sampled. To do this, first sample *s*NL_i (*i* = 1,...,*k*) from the estimated normal distribution of log(-log) transformed one-year survival. The mean of this distribution is estimated as log(-log(1-M_NL_)) and the standard deviation by log(-log(1-MNL_UB))- log(-log(MNL_LB)) divided by 2×1.96. The *s*NL_i are then back transformed to obtain deviates of survival *S*NL_i = exp(-exp(*s*NL_i)) and, hence, of mortality *M*NL_i = (1- *S*NL_i).
   2. Sample *k* deviates *r*_i_ (*i* = 1,...,*k*) of the proportion of lost to follow-up. To do this, sample deviates *NL_i* (*i* = 1,...,*k*) of numbers lost to follow-up from the binomial distribution with parameters *n* = *N_R_* and *p* = *N_L_ /N_R_*, where *N_R_* and *N_L_* are the number of eligible patients and the number of patients lost to follow-up, respectively. The *r*_i_ are obtained from *r*_i_ = *NL_i /N_R_*.
   3. Sample *k* deviates *M*L_i (*i* = 1,...,*k*) of one-year mortality among patients lost to follow-up analogously to a) using user input for M_L_, ML_UB and ML_LB.
   4. Obtain *k* deviates of corrected one-year mortality *M*C_i =(1- *r_i_*) *MNL_i* + *r_i_* *ML_i* (*i* = 1,...,*k*).
   5. The corrected estimate of one-year mortality and the 95%CI lower and upper bounds which are given as output correspond to the 50 (median), 2.5 and 97.5 percentiles of the sampled deviates *M*C_i (*i* = 1,...,*k*).
2. **Meta method**

This procedure is identical to described for 1) Tracing method, with the exception that step c) is replaced by the following:

For each deviate *r*_i_ (*i* = 1,...,*k*) obtained in b) sample a deviate *ML_i* of one-year mortality among patients lost to follow-up using the meta regression. To do this, sample deviates *mL_i* from the normal distribution for the logit of mortality among patients lost to follow-up for a programme with proportion of lost to follow-up equal to *r*_i_ taking into account the sampling variability of the meta regression parameters. The mean of this distribution is estimated as *a* + *br*_i_ and the variance as *ω_aa_* + *ω_ab_r*_i_ + *ω_bb_r*_i_^2^ +*τ*^2^ where *a* and *b* are the constant and slope parameter, *ω_aa_*, *ω_bb_* and *ω_ab_* are the variances and covariance of these parameters respectively, and *τ*^2^ is the between programme variance from the fitted meta regression.[2] The *ML_i* are then obtained from *ML_i* = exp(*mL_i*) / (1+ exp(*mL_i*)).

References

1. Kalbfleisch, J. D. and Prentice, R. L. (2002) The Statistical Analysis of Failure Time Data. New York, N.Y.: John Wiley and Sons.

2. Brinkhof MW, Pujades-Rodriguez M, Egger M (2009) Mortality of patients lost to follow-up in antiretroviral treatment programmes in resource-limited settings: systematic review and meta-analysis. PLoS One 4: e5790.
